# Desirable but not feasible: Measures and interventions to promote early childhood health and development in marginalized Roma communities in Slovakia

**DOI:** 10.3389/fpubh.2022.942550

**Published:** 2022-10-05

**Authors:** Daniela Filakovska Bobakova, Shoshana Chovan, Lucia Bosakova, Richard Koky, Marlou L. A. de Kroon, Zuzana Dankulincova Veselska

**Affiliations:** ^1^Department of Health Psychology and Research Methodology, Faculty of Medicine, PJ Safarik University, Kosice, Slovakia; ^2^Olomouc University Social Health Institute, Palacky University in Olomouc, Olomouc, Czechia; ^3^Department of Community and Occupational Medicine, University Medical Center Groningen, University of Groningen, Groningen, Netherlands; ^4^Department of Public Health and Primary Care, Centre for Environment and Health, Catholic University Leuven, Leuven, Belgium

**Keywords:** marginalized Roma communities, early childhood, health, development, equity, measures, interventions, feasibility

## Abstract

Roma are the largest and most disadvantaged ethnic minority in Europe often facing generational poverty, and limited access to education, employment, housing, and various types of services. Despite many international and national initiatives, children from marginalized Roma communities face multiple risks and are being disadvantaged as early as from conception onward. We, therefore, aimed to identify measures and/or interventions targeting equity in early childhood health and development in marginalized Roma communities which implementation is considered to be urgent but not feasible. We used a group concept mapping approach—a participatory mixed research method—and involved 40 experts and professionals from research, policy and practice. From 90 measures and interventions proposed to achieve early childhood equity for children from marginalized Roma communities, 23 measures were identified as urgent but not feasible. These concerned particularly measures and interventions targeting living conditions (including access to income, access to housing, and basic infrastructure for families) and public resources for instrumental support (covering mainly topics related to financial and institutional frameworks). Our results reflect the most pressing issues in the area of equality, inclusion and participation of Roma and expose barriers to implementation which are likely to arise from public and political discourses perpetrating a negative image of Roma, constructing them as less deserving. Measures to overcome persistent prejudices against Roma need to be implemented along with the measures targeting equity in early childhood health and development.

## Introduction

### Roma in Europe and Slovakia

Roma are the largest and most disadvantaged ethnic minority in Europe as well as in Slovakia. There are approximately 10 to 12 million Roma in Europe (est. 6 million are citizens or residents of the EU) and the highest share of the Roma population can be found in Central and Eastern European countries such as Bulgaria (10.3%), Slovakia (9.1%), Romania (8.3%), and Hungary (7%). A significant Roma population can be found also in Spain (1). According to a 2016 survey conducted in nine EU member states including Slovakia, about 80% of Roma live below the poverty line, 30% of Roma live in a household without tap water inside the dwelling or live in dwellings with a leaking roof, damp walls or other problems with the housing structure (2).

Out of approximately 440,000 Roma living in Slovakia, more than half live in so-called marginalized Roma communities (MRCs) located mostly on the outskirts or outside of municipalities (3). According to the Atlas of Roma communities, there are more than 1,000 MRCs (3) characterized by spatial and social distance from the majority population. Roma living in MRCs often experience generational poverty, limited access to education (including pre-primary education), employment, housing, and various types of services including health care, early childhood education, and care (ECEC) programs or nurseries (4–8). According to the findings of the EU SILCK MRK conducted in Slovakia 2020, 87% of households in MRCs are at risk of poverty, 52% face serious material deprivation, 58% have inadequate housing, and 88% of Roma living in MRCs live in overcrowded households (9).

### Early childhood health and development in MRCs

The above-mentioned unfavorable circumstances are reflected in health inequities between Roma and the majority population, which begin early—even before childbirth—and continue to develop during the life course (6, 10). Social circumstances, experiences, and relationships shape and reshape brain and body development, especially in early childhood (11). In the first 1,000 days of life counting from conception onwards, the rapid process of neurodevelopment takes place and the foundation is laid for further cognitive, socio-emotional, behavioral, personal, and language development as well as for health, with later impact on academic and professional achievement (12, 13).

Health inequalities between Roma children and children from the majority population in Slovakia are observable in multiple indicators such as neonatal mortality, the prevalence of Sudden infant death syndrome, the occurrence of infectious and parasitic diseases, as well as in the prevalence of hospitalizations (14–17). Although exposure to a wide range of social determinants of health and poorer health outcomes were found to be associated with early childhood development (18), data on early childhood development in Roma children are lacking. However, it is well-known that children, whose health and early childhood development are affected by contextual characteristics such as poverty, segregation, lack of stimulation, environmental risks, higher incidence of illnesses, malnutrition, and/or adverse childhood experiences, are in a disadvantaged position compared to their better-off peers (19). In marginalized Roma communities in Slovakia, children face multiple of these risks and are disadvantaged as early as from conception onward.

### Policies and interventions targeting MRCs

The most positive impact and largest financial returns are potentially generated by policies targeting vulnerable populations early in the developmental course (20, 21). Projects implemented on the national level, targeting MRCs, and funded from Structural funds such as National Project Field Social Work, National Project Community Centers or National Project Support for Pre-primary Education of Children from MRCs address early childhood only marginally (22). National Project Healthy Communities focuses on the inclusion of Roma in the area of health *via* health mediation. Activities of Roma health mediators concerning children aged 0–3 are focused mostly on ensuring participation in mandatory preventive examinations and vaccinations (23). Thus, in Slovakia, no systematic public ECEC programs targeting children aged 0–3 and fostering parenting competencies are present at the national level (22). Early childhood care services are accessible only to children with disability and even their access to these services is limited, meaning that these services are provided only to a fraction of disadvantaged children. Moreover, the accessibility and quality of early childhood care services are negatively affected by the poor cooperation of responsible ministries and local governments (24). Employment and labor market policies fail to address the unemployment trap and the ability of socially excluded long-term unemployed Roma to find a job in the open labor market (22). In 2018, Act on Social Economy and Social Enterprises was adopted, setting conditions for establishing social enterprises and providing a range of support mechanisms. However, its impact was not assessed yet (22). Concerning housing, the state gradually created a system of supportive economic tools for housing development and invested substantial financial resources, yet the housing situation of Roma continues to be unfavorable (25).

Whereas state policies concerning employment, housing, and early childhood health and development fail to address the disadvantages of the Roma living in MRCs, the non-profit sector seeks to compensate. However, local-level initiatives of non-governmental organizations (NGOs), although considered to be successful, have not always gained public or political approval and were not scaled up to the national level (9). Currently, only several intervention projects focusing on early childhood health and development are running in MRCs in Slovakia (5). Examples of such projects are projects OMAMA (26) targeting psychomotor development of children aged 0 to 5 and Mission 1,000 (27) targeting health and care for pregnant women and children from conception up to 3 years of age. Both projects stand on the same philosophy. Women living in MRCs are hired and intensively trained to deliver interventions mostly directly in the households of families with children (26, 27). These are newer, developing, and gradually expanding projects in both cases. They are currently only able to cover the needs of a fraction of children from MRCs, whereas the need far exceeds the current capacity of the projects (24). Other local projects targeting housing consist of the self-help construction of detached houses with the use of micro-loans and have been organized by the non-governmental sector on a small scale (24). An example of such a project is a joint venture of three NGOs and the commercial bank called DOM.ov which is present in 8 MRCs (22). Concerning employment, individual initiatives of commercial subjects responding to the current shortage of labor have started to appear. NGOs and foundations play an important role in the assistance and support of these initiatives (22).

In other European countries, the situation seems to be in many aspects similar. According to a meta-evaluation of 140 interventions for Roma inclusion from 30 countries, some interventions have been working for a long time and are still not necessarily sustainable due to the lack of long-term engagement of public institutions and financial support (28). Several interventions in the area of ECEC or health care presented promising outcomes, however, these interventions focused on older—preschool-aged—children or whole families (28). Many community-based early childhood programmes serving young Roma children have not been comprehensively documented and evaluated (29).

Despite all these initiatives, the desired change has not been achieved yet. Both, European and national level policy recommendations set the direction (30–34) and previous research accumulated knowledge on measures and interventions aimed at reducing the inequities between children from marginalized Roma communities and children from the majority population [see for example (29, 35–38)]. Many intended measures and successful small-scale projects or initiatives with good potential for positive results, including those focused on early childhood health and development, face significant barriers to implementation and up-scaling not only in Slovakia but also in other Central and Eastern European countries (5, 29). These barriers might include a lack of funding, continuity, capacities, and more (9). However, the evidence is scarce on which types of measures and interventions might be desirable but not feasible, influenced by possible barriers endangering their successful implementation and expected outcomes. The only study including perspectives of key stakeholders focusing on how to make healthy early childhood development more likely in MRC aimed to identify priority measures based on their urgency and feasibility (30). To facilitate the discussion at the national and international level, the way forward might be the identification of barriers and interpretation of low feasibility of measures and interventions perceived by relevant stakeholders as necessary to achieve equity in early childhood health and development for marginalized Roma children.

Therefore, this study aimed to identify and discuss a group of measures and/or interventions targeting equity in early childhood health and development in marginalized Roma communities which implementation is considered by professionals from different fields working with marginalized Roma communities to be urgent but not feasible.

## Materials and methods

### Design

We used a group concept mapping (GCM) approach to structure perceptions of various stakeholders and experts from different fields on how to promote early childhood health and development in MRCs. GCM uses a participatory approach and combines the qualitative and quantitative methodology of data collection and analysis (39) for assessing how study participants cluster their conceptual assessment of a particular topic (40). The study was approved by the Ethical Committee of the Faculty of Medicine at PJ Safarik University under the number 12N/2020.

### Sample

To capture a wide spectrum of perspectives, participants of multiple expertise were recruited. Purposive sampling was used to involve professionals from different backgrounds with a deep understanding of the various determinants influencing healthy development and the sources of early adversities. Sample selection was informed by the theoretical Biodevelopmental framework for understanding the origins of disparities in learning, behavior, and health (41) and targeted professionals working with marginalized Roma communities from Slovakia from both public and non-governmental sectors and of different levels of work hierarchy across these categories: health care providers, social workers, community center workers, early childhood educators, special educators, health mediators, experts in early childhood development and policymakers. Both Roma and non-Roma experts/professionals were included. Out of 79 professionals invited to participate in the study, 54 agreed to take part (mean age 42.5; 77.5% women) and completed the *brainstorming phase* of the study as described below. The final sample for the further *sorting-rating phase* consisted of 40 participants which is, according to GCM methodology, a sufficient number to meet the statistical requirements for valid and reliable results (42). The background characteristics of this sample can be found in Table 1.

**Table 1:** Background characteristics of the sample.

	***N***	**%**
**Gender**		
Men	9	22.5
Women	31	77.5
**Education**		
Elementary	1	2.5
Secondary	6	15.0
University	33	82.5
**Working in direct contact with the target population**		
Yes	34	85.0
No	6	15.0

### Procedure and analyses

The study was conducted between June 2020 and March 2021. The procedure consisted of five steps (phases): preparation, brainstorming, sorting and rating, analysis, and interpretation (40, 43).

In the *preparation phase*, the focal question: “What needs to be done to equalize the chances for healthy early childhood development of children from marginalized Roma communities with the majority population?” was formulated. The potential participants were identified and contacted, and the schedule of the project was determined. Conducting the study online, using conference calls and the groupwisdom™ platform for each concept-mapping activity was agreed and the aim of the study and the GCM procedure was explained and discussed during the initial conference call attended by the research team and participants.

The *brainstorming phase* was performed on the groupwisdom™ online platform. Participants who signed informed consent forms were encouraged to brainstorm as many ideas and measures to answer the focal question as possible. This phase was anonymous to protect participants from potential power relations. Initial proposals produced by the participants in the brainstorming phase were reviewed and synthesized by an expert group consisting of 6 researchers. First, semantically similar proposals were merged and proposals holding more than a single measure/intervention were split so that the redundant and overlapping concepts were removed and reduced, parsimonious set of statements was created. Second, items that could not be a policy issue, items that described a problem rather than a solution or a measure targeting the problem, and items targeting different age groups than that specified in the focal question were removed. A final set of proposals also known as a master list (40) was then once again sent to the participants for commenting and clarification.

In the *sorting-rating phase*, the participants were asked to sort the proposals from the master list into groups or piles of similarly themed statements and to name each of the groups based on what they view as the unifying topic or content of each group. Subsequently, they were asked to rate these proposals according to two selected domains of interest, i.e., urgency and feasibility, on a 7-point Likert scale (1—not urgent/not feasible, 7—very urgent/very feasible). Sorting and rating were performed using the groupwisdom™ platform.

The *analytic phase* focused on final data categorization. A quality review was performed, to exclude participants who did not follow sorting and/or rating guidelines (did not complete at least 75% of the task, or gave negligent answers). Statistical analysis of the data was performed using the groupwisdom™ platform. Sorting data were analyzed using multidimensional scaling and hierarchical cluster analysis to generate a cluster solution where proposals for measures and interventions were aggregated into clusters based on the number of times participants grouped them together (40). The findings were discussed by the research team and the resulting cluster solution was chosen based on the most consistent cluster map. Cluster labels were discussed and subtopics were identified in each of the clusters. Next, rating data were analyzed and a Go-Zone map, i.e., an X–Y graph which compares items across two rating criteria (urgency and feasibility) and is divided into quadrants above and below the mean value of each rating variable (44) was produced. The model fit was checked using the stress-index i.e., the degree to which the distances on the map are discrepant from the values in the input similarity matrix; a high stress-index value indicates a greater discrepancy (i.e., the map does not represent the input data well) (40, 45).

In the *interpretation phase*, the final cluster solution and cluster labels proposed by the research team were sent to the participants to gain their feedback on these results and their interpretation of the resulting maps. These outcomes were further discussed during the workshop with the interpretation group consisting of 17 selected participants of whom most participated in previous steps. This group of participants consisted of representatives of the state and non-profit sectors covering areas of expertise in the field of health, social and preventive counseling.

## Results

### Sorting and rating of proposed measures and interventions

Participants proposed 178 measures and interventions targeting early childhood health and development in MRCs. As described in the Methods section, these were reviewed and condensed by the expert group into 90 distinct proposals for measures and interventions (Master list) and sent to participants for sorting and rating. Participants sorted proposed measures and interventions into 3 to 13 thematic groups. The expert group chose and approved the final 4-cluster solution and identified subtopics contained in these clusters (Table 2). The stress index of 0.1916 indicates a strong fit between the cluster map and the data (46).

**Table 2:** Clusters of measures and interventions targeting early childhood health and development in MRCs in Slovakia according to professionals working with the target population.

**Cluster label**	**Subtopics**
1. Public resources for instrumental support	1. Financial and institutional frameworks 2. Tools for instrumental support
2. Enhancement of living conditions	1. Access to income 2. Access to housing 3. Access to basic infrastructure
3. Quality and accessibility of health care	1. Prenatal care 2. Perinatal care 3. Postnatal care 4. Pediatric care 5. Reproductive health 6. Field health care
4. Community interventions focused on the transfer of cultural capital	1. Who should be educated 2. By whom 3. Where should this education take place 4. How should the education occur 5. What should be the content of the education

Based on participants' rating of urgency and feasibility, 23 proposals for particular measures and interventions were identified as urgent but not feasible (Table 3), indicating that these desirable proposals might face barriers to implementation. Most of these proposals belong to Cluster 1 (*Public resources for instrumental support*) and Cluster 2 (*Enhancement of living conditions*), the latter being the least feasible group of measures and interventions on average. The most urgent proposals with low feasibility are targeting the employment of Roma (proposals 10, 11). The least feasible measures with values being lower than the median concern enhancement of living conditions, specifically, construction of social housing (proposal 17), legislative change removing obstacles to drinking water supplies (proposal 14), and improving access to income for the Roma population by introducing sheltered employment programs in companies, training on the part of employers, creating tax benefits and incentive bonuses (proposal 9). Another six proposals rated as urgent but not feasible belong to Cluster 3 (*Quality and accessibility of health care*) and Cluster 4 (*Community interventions focused on the transfer of cultural capital*) and concern for example outreach healthcare, preparatory classes for children before entering preschool or mandatory educational activities for underage mothers.

**Table 3:** List of urgent but not feasible measures and interventions targeting early childhood health and development in MRCs in Slovakia according to professionals working with the target population.

**Proposals by cluster**	**Average rating**	
	**Urgency^a^**	**Feasibility^b^**
**Cluster 1** ***Public resources for instrumental support*****—topics related to** ***Financial and institutional frameworks*** **and** ***Tools for instrumental support***.		
1. Establish an institute of Roma assistant (at least available by telephone) and create a network/database of such assistants, that can be contacted by any institution, where communication is an essential condition for successful intervention—preschools and primary schools, first contact clinics, foster home facilities, asylum facilities, municipal and state police, etc.	5.2750	4.0278
2. The state should increase the overall allocation of funds to education, financially support the creation and operation of programs for children younger than 3 years.	5.6250	3.6667
3. Ensure state-paid Roma field assistants in each MRC, so that the child is caught as soon as he arrives from the maternity ward and subsequently monitored, that the parents are monitored, to see if the child has a health insurance company, birth certificate, district pediatrician, preventive examinations. Implement family supervision.	5.5526	3.8056
4. Create a grant program to support non-governmental organizations providing early childhood care in the field.	5.1316	3.9444
5. Financially and institutionally support the Early Intervention Centers and create a regionally accessible network of centers that are able to provide interventions in the field and specifically in the MRC (network expansion, strengthening human resources, technical and material security, and strategies for further development).	5.1750	3.1944
6. Guarantee a legal right to access early childhood care services for all at-risk children (health, social).	5.2500	3.9722
7. Develop a supra-ministerial strategy for early childhood education and care as a cross-sectoral and multidisciplinary system (in reconciling work and family life, health, social services, and education), including standards for early childhood care provision, a monitoring system of quality, and a funding system.	5.3750	3.6111
8. Ensure the continuity of financing early care programs from the state budget within the social policy of the state, e.g., through municipal enterprises or the Office of the Government Plenipotentiary for the Roma Community.	5.5789	3.7143
**Cluster 2** ***Enhancement of living conditions*****—topics related to** ***Access to income, Access to housing**,* **and** ***Access to the basic infrastructure***.		
9. Improve access to income for the Roma population (by introducing sheltered employment programs in companies, training on the part of employers, creating tax benefits and incentive bonuses).	5.4103	2.9722
10. Increase Roma employment through social enterprises.	6.0000	4.0000
11. Create as many job opportunities as possible for parents so that they can raise their standard of living.	5.7750	3.2222
12. Employ women from communities, that provide early childhood care, on full-time contracts.	5.5385	3.9722
13. Negotiate with mayors about enabling access to drinking water, heat, garbage collection, and so on.	5.4103	3.9167
14. Special legislation on access to drinking water and legislative removal of obstacles to the management of infrastructure for water networks, including the law on the removal of all obstacles to the supply of drinking water to all households, regardless of whether it is a legal or illegal building.	5.3590	2.9118
15. Ensure the cooperation of relevant actors (municipality, self-governing region, private sector) in building infrastructure (water, sewerage, utilities, roads, and sidewalks).	5.6410	3.3611
16. To support the motivation of municipalities to address the issue of housing in the MRC from the government level.	5.6250	3.4571
17. Ensure the construction of social housing.	5.1250	2.8529
**Cluster 3** ***Quality and accessibility of health care*****—topics related to** ***Prenatal care, Perinatal care, Postnatal care, Pediatric care, Reproductive health, and Field health care***.		
18. Provide multidisciplinary support immediately after childbirth (nurse together with a doctor, social worker, field worker).	5.2308	3.7714
19. Improve the availability of prenatal care by requiring the corresponding GP to be obliged to take every woman from his or her district into care. Provide assistance of field workers in this process so that the woman cannot be rejected.	5.5000	4.0556
20. Implement a system of field pediatric and nursing care.	5.3500	3.3333
21. Implement a system of field nurses, who would visit mothers in their homes during the postpartum period, teach and help with individual aspects of newborn care.	5.7000	3.5714
**Cluster 4** ***Community interventions focused on the transfer of cultural capital*****—topics related to** ***Who should be educated, By whom, Where should this education take place, How should the education occur*** **and** ***What should be the content of the education***.		
22. Create preparatory classes for children who are younger (they could be created, for example, at the level of community centers), where trained workers would work with children and prepare them for entering preschool.	5.1795	3.8056
23. Mandatory educational activities (on child development and care, responsible parenting…) for all underage mothers, regardless of ethnicity during pregnancy and after childbirth.	5.5385	3.7500

^a^Urgency (1 = not urgent; 7 = very urgent): Scale [3.2500]–[6.1795]; Median = 3.08975; *n* = 40.

^b^Feasibility (1 = not feasible; 7 = very feasible): Scale [2.4000]–[6.0000]; Median = 3; *n* = 36.

### Interpretation of measurers and interventions identified as urgent but not feasible

#### Public resources for instrumental support

The most discussed topic during the interpretation workshop with a group of participants was the role of the state in ensuring the financial and legislative conditions for the continuity, quality and upscaling of programs focused on health and healthy development in early childhood which are currently implemented mostly by NGOs and/or financed mostly from structural funds. The failure of the state in the case of financing was seen in long-lasting passivity, reluctance to take responsibility and reliance of the state on the non-profit sector. Representatives of the non-profit sector pointed out their “*struggles with fighting for the sustainability of activities*” and that they would welcome the creation of grant schemes by the state. A barrier was seen in relieving the responsibility of individual ministries, as most of the activities and services provided in marginalized communities are cross-sectional, and it is difficult to define which ministry shall be responsible for which services. At the same time, the need to financially support the strengthening of the personal capacities of existing service providers was emphasized.

According to the participants, the above-mentioned barriers related to the financial responsibility of the state are closely interconnected to legislative conditions. Regarding the role of the state in creating legislative conditions, the need for a supra-ministerial strategy was emphasized. The biggest problem in this area at present, according to the participants in the interpretation group, is the lack of coordination of services provided in individual areas (health, social and preventive counseling) and a clear definition of competencies and activities falling under individual ministries (Ministry of Health, Ministry of Labor, Social Affairs and Family and Ministry of Education, Science, Research and Sport). Participants emphasized that the state should thus guarantee the quality of education, continuity, networking and cooperation of service providers. The need for supra-ministerial workplaces at a higher level of management and multidisciplinary teams operating in the field was articulated. Without a supra-ministerial approach, providers “*face the problem that many families fall through the network of support*” provided by the existing network of services, and many families are left behind.

Moreover, as participants indicated, if the nationwide operation of some programs in the field of early childhood should come under the responsibility of the state, the barrier could be the setting of formal quality criteria and qualification criteria for people working in the field. This barrier relates to the need to apply participatory approaches and to employ and educate people from MRCs to provide health, social and preventive counseling services directly in communities, to cooperate with respective professionals providing their services in various centers and to build the bridges to bring those services closer to the people in need.

#### Enhancement of living conditions

Enhancement of living conditions was seen as a crucial precondition for the successful implementation of all kinds of educational and awareness-raising interventions. Implementation of such interventions in the environment of indecent living conditions of families characterized by unsuitable housing, lack of running drinking water and unstable income, was seen as “*cherries on a cake that does not exist*”. The need for the active involvement of local governments and higher territorial units was emphasized. According to the participants, this may be possible in the next programming period by applying for the Structural Funds, and from the position of the Office of the Plenipotentiary of the Government of the Slovak Republic for Roma Communities, this is one of the priorities.

#### Quality and accessibility of health care

According to the interpretation group, the barrier in health care lies in the generation of primary care providers in retirement age, which will be difficult to replace, and in the overcrowded capacities of primary health care providers, who often take care of 2,500–3,000 children from the catchment areas of MRCs. As participants suggested, proposed measures related to multidisciplinary support of mothers and implementation of field pediatric and nursing care “*should be implemented on the national level under the responsibility of the Ministry of Health*” but might face the same barriers as mentioned within the cluster of *Public resources for instrumental support*. A lack of financial support and barriers posed by current legislative conditions not only hinder the implementation of these interventions but also those initiatives which are legally possible but the administrative burden of which, arising from legislation, makes them unfeasible. Participants in the interpretation group mentioned an example of providing health care in two outpatient departments by one health care provider. They see this as much needed to bring primary health care closer to people, but rather discouraging for health care providers, as it is burdened by a complicated administrative process required by health insurance companies and the state.

#### Community interventions focused on the transfer of cultural capital

Although most of the proposed measures and interventions from this cluster which are highly focused on educational activities were seen as both urgent and feasible, those seen as less feasible would again require legislative changes. For example, using community centres for preparatory classes for children before entering preschool is currently not possible due to restrictions posed by current legislation. The activities of community centres are defined by law, and it is not possible to go beyond what the law defines. Apparently, everything that requires legislative change is seen as unfeasible. When discussing educational activities for parents and children targeting early childhood health and development with the interpretation group, the question of the legitimacy of the measures and interventions introduced in MRCs was raised. According to the members of the interpretation group, we need to sensitively define the goals we want to achieve and what we want to offer to families “*without colonizing them with visions and lifestyles*” of the privileged majority population and without the risk that “*we will need to apologize 30 years later for what we have done*”. The goals highlighted by the interpretation group included the goal of relieving children of the toxic stress they experience in generational poverty, minimizing the risks and making it possible for these children to be more successful later at school and consequently later in life. Following that, the interpretation group also emphasized the need to evaluate existing initiatives and to conduct a participatory needs assessment to design future measures and interventions.

The overarching topic discussed within the interpretation group was the need to change the social discourse about marginalized Roma. A media campaign which was rated as both urgent and feasible was not seen as the most powerful tool to do so by the interpretation group. The need for education at all levels, from children to professionals, which could pave the way for people to “*positively perceive and accept diversity*” and to break down stereotypes, was articulated. Also, the media image of Roma needs to change, as the media nowadays tend to choose and promote negative, stereotyping messages about marginalized Roma.

## Discussion

This study aimed to identify a group of measures and/or interventions targeting equity in early childhood health and development in MRCs which implementation is considered by professionals from different fields working with MRCs to be urgent but not feasible. From 90 measures and interventions proposed to achieve early childhood equity for children from MRCs, 23 measures were identified as desirable but not feasible. The least feasible urgent measures are targeting living conditions and covering topics such as access to income, access to housing, and basic infrastructure (drinking water, sewerage, utilities, roads, and sidewalks) for families. A substantial number of urgent but not feasible measures concern also public resources for instrumental support and cover mainly topics related to financial and institutional frameworks (e.g., financial and institutional support of ECEC services and Early intervention centers, legal right to access them) but also overarching target to develop a supra-ministerial strategy for ECEC. Previously published recommendations on how to achieve equity in early childhood health and development can be found mostly in policy papers and reports which are using available data describing the problem itself rather than asking directly and participatively about the solution of an existing problem as done in our study. In addition to that, most of the previously published work focus on Roma children in preschool age [see for example (29, 36–38)] and studies on children aged 0–3 are scarce thus children in this crucial age period seem to be left behind in terms of both—accessible interventions and data [see for example (8, 28, 47)].

The highest number of urgent but not feasible measures and interventions was found in Cluster 2 related to the enhancement of living conditions. Also, three urgent measures rated as the least feasible are from this cluster and target construction of social housing, access to drinking water by removing legal obstacles, and access to income through sheltered employment programs, training for employees, and benefits for employers. These measures are targeting elementary needs necessary for laying the basis of decent living conditions rather than targeting (a healthy) early childhood as such. This is supported by previous research in which housing was found to be significantly associated with early childhood health and development (52, 53). Importantly, the direct and sustainable effects of other measures targeting specifically early childhood such as ECEC might be limited unless these fundamental needs are met (48–50). Moreover, the social gradient in access to and use of early childhood education and care services causes children from disadvantaged backgrounds to use such services to a lesser extent (29, 54). Access to income, access to housing, and the basic infrastructure for families are considered to be fundamental for combating child poverty and achieving equity in health and healthy development in early childhood in children from MRCs (34, 54–56). However, due to the diversity of Roma populations across Europe and the differences in national economic conditions and legislation, no universal or simple solutions can be applied to bring broad and sustainable changes in the contexts in which children from marginalized Roma communities grow up (29). Thus, on one hand, measures and interventions should be adapted to the context but on the other hand, they should also aim to better this context to the degree in which children would be able to thrive and benefit from all other support and services provided to them and their families.

Another group of measures perceived as urgent but not feasible is related particularly to financial and institutional frameworks. The overarching proposal within this group of measures seems to be the development of a supra-ministerial strategy for early childhood education and care as a cross-sectoral and multidisciplinary system. This includes standards for early childhood care provision, a monitoring system of quality, and a funding system. Other related proposals are associated with ensuring financial stability and continuity of health mediation and early childhood intervention programs, implying that this should not depend on grant schemes but on a stable and fundamental contribution from the state budget. Funding was identified as an important obstacle in similar programmes in Europe not funded by the government (38). Using European funds and analogous funding opportunities as the main, and in some cases, the only funding of Slovakia's key programmes focusing on Roma inclusion is burdened by delays, complicated application and administration, and associated hesitancy of potential recipients to apply for it (5). This might be one of the barriers to the implementation of otherwise successful interventions or strategies with good potential for positive results. Guaranteeing stable financing from the state budget would ease the burden of raising funds for the operation of intervention programs and create space and opportunities to focus on quality improvement and capacity building instead of struggling with running costs.

The body of evidence on the root causes of inequities is robust enough and known long enough to combat them (10), however, there seems to be a gap between knowledge and action as “the evidence on its own does not provide a complete recipe for success, nor an imperative for action” (51). Little or no progress was made in the elimination of inequities when it comes to marginalized Roma communities (6, 57), although policy, legal and funding instruments have been aligned and mobilized since 2011 when the European Commission (58) and Council of Europe (59) called for such action. Many European states have adopted National Roma Integration Strategies focused on four key areas (education, employment, housing and health). On one hand, closing the gap in a single generation and reversing the effect of centuries of continuous and systematic exclusion and oppression seems to be unrealistic (60), on the other hand, our society is far from addressing one of the fundamental root causes—anti-Roma racism—deeply rooted across societal structures and penetrating from the public into the political discourses (61–63). Combating anti-Roma racism is finally seen as a priority area of the EU Roma strategic framework for equality, inclusion and participation (64), nevertheless, we still seem to be at the beginning of this journey as less conspicuous forms of modern racism may be hard to challenge (62). The low feasibility of measures targeting living conditions and using public resources for instrumental support is likely to be caused by one common factor, which is the low political will to implement measures that are considered to be unpopular due to high expected investments from public resources toward a group perceived as less deserving. Moreover, political will is among the essential ingredients for policy action but in some countries, there is still a lot to be done in making the case to policymakers about the need to tackle health inequities (51). Low political will can explain low investments (24), which essentially leads to the infeasibility to implement measures that are urgently needed. Positive measures targeting people living in MRCs are often perceived as “favors granted to them rather than necessary actions to safeguard their equality of rights” (64). The public discourse in Europe perpetrating stereotypical, racist, hateful, and discriminatory views about Roma (65) is reflected in excluding Roma communities from national solidarity (64). This causes policies targeting Roma unlikely to succeed without measures to overcome persistent prejudices against them in the majority society (29, 55). However, many policies and programmes fail to sufficiently address combating anti-Roma racism (65). Findings from our study contribute to the ongoing professional and political discussions on public health policies targeting equity in early childhood health and development and shed some light on measures and interventions perceived as desirable but not feasible due to barriers to implementation.

### Strengths and limitations

The main strength of this study can be seen in the size and quality of the sample involving a range of stakeholders from research, policy, and practice with different but mutually enriching perspectives on the studied topic. This sample composition allowed for the conceptualization of relevant proposals for measures and interventions targeting early childhood health and development in MRCs. Although out of the initial 54 participants 14 participants dropped out between the brainstorming and the sorting-rating phase of the study, GCM methodology takes some losses of participants between the steps into account without bias being likely (42). Moreover, the methodology of GCM might be prone to social desirability and subjectivity typical for qualitative studies. To cope with social desirability but also with the potential effect of mutual power relations between participants, we made the brainstorming phase anonymous. By using a participatory approach and discussing each step of the study with the participants and within the wider research team, all decisions were made jointly and the “four-eye principle” was applied. Another limitation of this study may be seen in the purposive sample consisting mostly of women. The expert field concerning the topic of interest is limited and feminized in Slovakia, nevertheless, the sampling was informed by a Biodevelopmental framework (41) to ensure a rich spectrum of viewpoints.

### Implications

Allocating financial resources from the state budget might promote the quality and capacity of promising initiatives and ensure their continuity. Implementation of measures and interventions expecting and involving investments from the state budget requires the audacity of policymakers not to adapt political will to discourses questioning and rejecting equity and human rights for groups left behind and excluded from society. Combining the resources of the governments with European funding opportunities needs to be accompanied by tools to support potential recipients in the application and administration of such projects.

The need for intra-sectoral cooperation and multisectoral programming requires commitment and strategic planning blurring the borderlines of agendas of particular ministries. Broad and sustainable changes aiming at social inclusion will need to take into account not only community-based interventions in the area of ECEC services (4) but also measures that combat poverty, promote tolerance toward marginalized Roma communities, and address prejudices and discrimination that are embedded across societal structures (29, 59, 65). Compliance with the UN Convention on the Rights of the Child should be ensured by the development of accountability procedures and mechanisms with a capacity to develop strategies for child rights enforcement (66, 67).

Discordance between the complicated policy processes and the efforts of advocates for the action on social determinants of health seems to pose a significant barrier to the implementation of desired measures and interventions (68). According to Carey and Crammond, “the type of evidence which exists, the way it is framed in policy proposals, and the way it is presented by researchers and advocates all reflect a belief that providing enough evidence of the problem will be sufficient to spur political action” (68). There is still a lot to be learnt on how to gain political traction and hold politicians' interest long enough to be turned into action. Moreover, the inability of states to overcome inequities can be seen as a systemic, institutional and structural form or manifestation of anti-Roma racism which is the most important barrier to Roma community empowerment and inclusion (66). States play important role in producing and reproducing oppressive practices and norms (68). Given these circumstances ability of states to take the lead in addressing inequalities is limited, thus the involvement of other actors seems to be necessary. Actors advocating on behalf of Roma, civil society organizations, health organizations, communities and academia working together toward mutual goals have the potential to fill the gap and contribute to changing societal discourse and build the social capital necessary to implement actions which currently seem to be unfeasible.

Future research should focus on the identification of contexts and mechanisms leading to the state in which some of the urgent measures as deemed unfeasible.

## Conclusion

Professionals from different fields of research, policy, and practice working with MRCs identified as urgent but not feasible, particularly measures and interventions targeting living conditions (including access to income, access to housing, and basic infrastructure for families) and public resources for instrumental support (covering mainly topics related to financial and institutional frameworks). On one hand, proposed measures and interventions rated as highly urgent reflect the most pressing issues in the area of equality, inclusion, and participation of Roma (32, 35), on the other hand, their low feasibility exposes barriers to implementation. These are likely to arise from public and political discourses perpetuating negative images of Roma (65), perceiving them as less deserving, causing investments toward marginalized Roma as unpopular, and resulting in excluding them from national solidarity (64).

## Data availability statement

The raw data supporting the conclusions of this article will be made available by the authors, without undue reservation.

## Ethics statement

The studies involving human participants were reviewed and approved by Ethical Committee of the Faculty of Medicine at PJ Safarik University under the number 12N/2020. The participants provided their written informed consent to participate in this study.

## Author contributions

LB and DF: conceptualization. LB: methodology. DF, RK, and SC: investigation and data curation, writing—review and editing. LB, SC, DF, and ZD: formal analysis. DF and SC: writing—original draft. ZD and MK: supervision. All authors contributed substantially to the study design, data analysis and interpretation of the findings.

## Funding

This work was supported by the Slovak Research and Development Agency under Contract no. APVV-19-0493 and by the Scientific Grant Agency of the Ministry of Education, Science, Research and Sport of the Slovak Republic and the Slovak Academy of Sciences, Reg. No. 1/0593/21.

## Conflict of interest

The authors declare that the research was conducted in the absence of any commercial or financial relationships that could be construed as a potential conflict of interest.

## Publisher's note

All claims expressed in this article are solely those of the authors and do not necessarily represent those of their affiliated organizations, or those of the publisher, the editors and the reviewers. Any product that may be evaluated in this article, or claim that may be made by its manufacturer, is not guaranteed or endorsed by the publisher.

## Abbreviations

ECEC: early childhood education and care
GCM: group concept mapping
MRCs: marginalized Roma communities.
